# Inhibiting the Appearance of Green Emission in Mixed Lead Halide Perovskite Nanocrystals for Pure Red Emission

**DOI:** 10.1021/acs.nanolett.4c01565

**Published:** 2024-09-23

**Authors:** Mutibah Alanazi, Ashley R. Marshall, Yincheng Liu, Jinwoo Kim, Shaoni Kar, Henry J. Snaith, Robert A. Taylor, Tristan Farrow

**Affiliations:** † Clarendon Laboratory, Department of Physics, University of Oxford, Parks Road, Oxford OX1 3PU, United Kingdom; ‡ Helio Display Materials Ltd., Wood Centre for Innovation, Oxford OX3 8SB, United Kingdom; § Institute of Materials Research and Engineering, Agency for Science, Technology and Research (A*STAR), 2 Fusionopolis Way, Singapore 138634; ∥, NEOM U, and Education, Research and Innovation Foundation, Tabuk 49643-9136, Saudi Arabia

**Keywords:** mixed lead halide perovskites, nanocrystals, thin films, phase segregation, encapsulation, halide sublimation

## Abstract

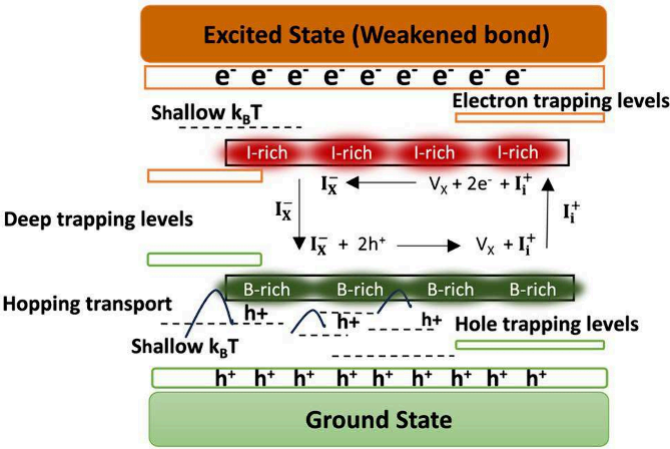

Mixed halide perovskites exhibit promising optoelectronic properties for next-generation light-emitting diodes due to their tunable emission wavelength that covers the entire visible light spectrum. However, these materials suffer from severe phase segregation under continuous illumination, making long-term stability for pure red emission a significant challenge. In this study, we present a comprehensive analysis of the role of halide oxidation in unbalanced ion migration (I/Br) within CsPbI_2_Br nanocrystals and thin films. We also introduce a new approach using cyclic olefin copolymer (COC) to encapsulate CsPbI_2_Br perovskite nanocrystals (PNCs), effectively suppressing ion migration by increasing the corresponding activation energy. Compared with that of unencapsulated samples, we observe a substantial reduction in phase separation under intense illumination in PNCs with a COC coating. Our findings show that COC enhances phase stability by passivating uncoordinated surface defects (Pb^2+^ and I^–^), increasing the formation energy of halide vacancies, improving the charge carrier lifetime, and reducing the nonradiative recombination density.

Metal halide perovskites are promising candidates for Pe light-emitting diodes (LEDs), with efficiencies exceeding 30% for green,^1^ 25% for red,^2,3^ and 10% for blue emission.^4^ Among the different types of perovskite emitters, all-inorganic lead halide perovskites offer tunable wavelengths, narrow line widths,^5^ high brightness, and high color purity,^6^ making them excellent candidates for next-generation PeLEDs. Mixed halide perovskites such as CsPbI_2_Br can achieve pure red emission by adjusting the Br:I halide ratio. However, these materials suffer from halide separation and compositional instability at photoabsorption or current injection.^7^ This leads to the spatial separation of halide species into distinct bandgap domains, causing a shift in the emission wavelength. In bulk films, iodide-rich regions form low-bandgap domains at grain boundaries, causing red-shifted emission. In contrast, the emission peak shifts toward blue in perovskite nanocrystals (PNCs), indicating bromine-rich emissive regions due to favorable bond breaking and iodide ion escape because of shorter Pb–Br bonds. Like bulk films, the iodide ion in PNCs migrates from the point of illumination/injection to adjacent sites due to Coulombic repulsion.^8^

Several theoretical models have been suggested to understand such phenomena on the basis of thermodynamic and kinetic perspectives.^9^ Among these models, the role of halide oxidation can rationally explain the observed halide separation in nanocrystal and thin film perovskites.^10^ Upon illumination, I/Br mixed perovskites undergo preferential iodide oxidation, resulting in local concentration gradients of oxidized products (e.g., I_2_ and I_3_^–^) and driving halide migration.^10−12^ Moreover, under continuous illumination, Pb^2+^ ions in perovskites are more likely to be reduced to metallic Pb^0^, decreasing the efficiency of the devices.

The propensity of perovskites to form aggregates in solution during synthesis can generate halide vacancy defects, creating localized states that capture injected carriers^13,14^ due to the ionic nature and dynamic surface coverage with ligands.^15−18^ This promotes self-doping and n-type behavior, resulting in inefficient exciton recombination. Moreover, the halide ions permeate the vacancies, accelerating ion migration due to weak van der Waals forces between Cs and X in the [PbX_6_]^4–^ octahedra.^19^

Several strategies have been used to prevent ion migration, including metal oxides, inorganic salts, a mixture of metal oxides and inorganic salts,^20,21^ metal–organic hybrids, and polymers.^22,23^ Among them, polymers are attractive due to their ability to undergo a transition from an extended coil state to a collapsed globule state when the solvent’s properties change. Polymers can act as protective layers in globular states, shielding NCs from their surroundings. In contrast, they partially expose the surface in coil states, allowing chemical reactions with the NCs and making them reactive. The transition from the globule to the coil phase of a polymer is influenced by the solvent used and can be carefully adjusted.^24^

Polymeric micelles were also used for the encapsulation of mixed halide perovskite nanocrystals^25−27^ and as templates for the synthesis of inorganic PNCs with stable surface coatings and low polydispersity.^28,29^ In addition, polymers with hydrophobic properties are often excellent for maintaining MHP NCs from polar solvents.^30,31^ According to a previous study, encapsulating CsPbBr_3_ NCs in matching hydrophobic macroscale polymeric matrices improved stability.^32^ The embedding of PNCs in hydrophobic bulk polymers, including poly(methyl methacrylate) (PMMA),^33,34^ polystyrene (PS),^35^ and poly(styrene-ethylene-butylene-styrene) (SEBS),^36^ has improved ambient stability and suppressed phase segregation. According to previous investigations, encapsulating CsPbBr_3_ NCs in matching hydrophobic macroscale polymeric matrices improved stability.^32^ Despite extensive research, PNCs remain insufficiently resistant to high fluid irradiation and high-volume ratios of polar liquids, and their scalable approaches are not widely accessible.

Here, we used cyclic olefin copolymer (COC) as an inexpensive and excellent insulating bulk polymer to encapsulate CsPbI_2_Br PNCs and their thin film counterparts. We observed massive suppression of phase separation in CsPbI_2_Br PNCs with a COC coating compared to that of the test sample without encapsulation, further achieving highly efficient pure red emission at 615 nm with stable PL spectra under intense illumination. The results demonstrated that COC could passivate Pb dangling bonds on the PNC surface, reducing the number of halide vacancies. Specifically, COC passivates the uncoordinated Pb^2+^-related defects at the surface of CsPbI_2_Br PNCs and increases the formation energy of halide vacancies (e.g., iodine vacancies), which, in turn, limits ion migration, prolongs the lifetime of charge carriers, and suppresses halide separation in perovskites.

We synthesized the CsPbI_2_Br PNCs and CsPbI_2_Br thin films and encapsulated them with COC by depositing perovskite precursors on glass substrates using spin-coating as described in Figure S1. The absorption and PL spectra of CsPbI_2_Br PNCs and CsPbI_2_Br thin films were measured under air conditions as shown in panels a and d of Figure 1. The exciton absorption and PL emission peaks of CsPbI_2_Br PNCs showed a slight blue shift compared to those of CsPbI_2_Br thin films. Compared with those of thin films, the exciton absorption and PL emission peaks of CsPbI_2_Br PNCs exhibited a marginal blue shift. The high-resolution transmission electron microscopy images in panels b and c of Figure 1 and Figure S1 revealed a homogeneous distribution with an average particle size of 11.3 nm for CsPbI_2_Br PNCs. The scanning electron microscope (SEM) image in panels e and f also confirmed a homogeneous distribution with an average particle size of 450 nm for CsPbI_2_Br thin films.

**Figure 1:**
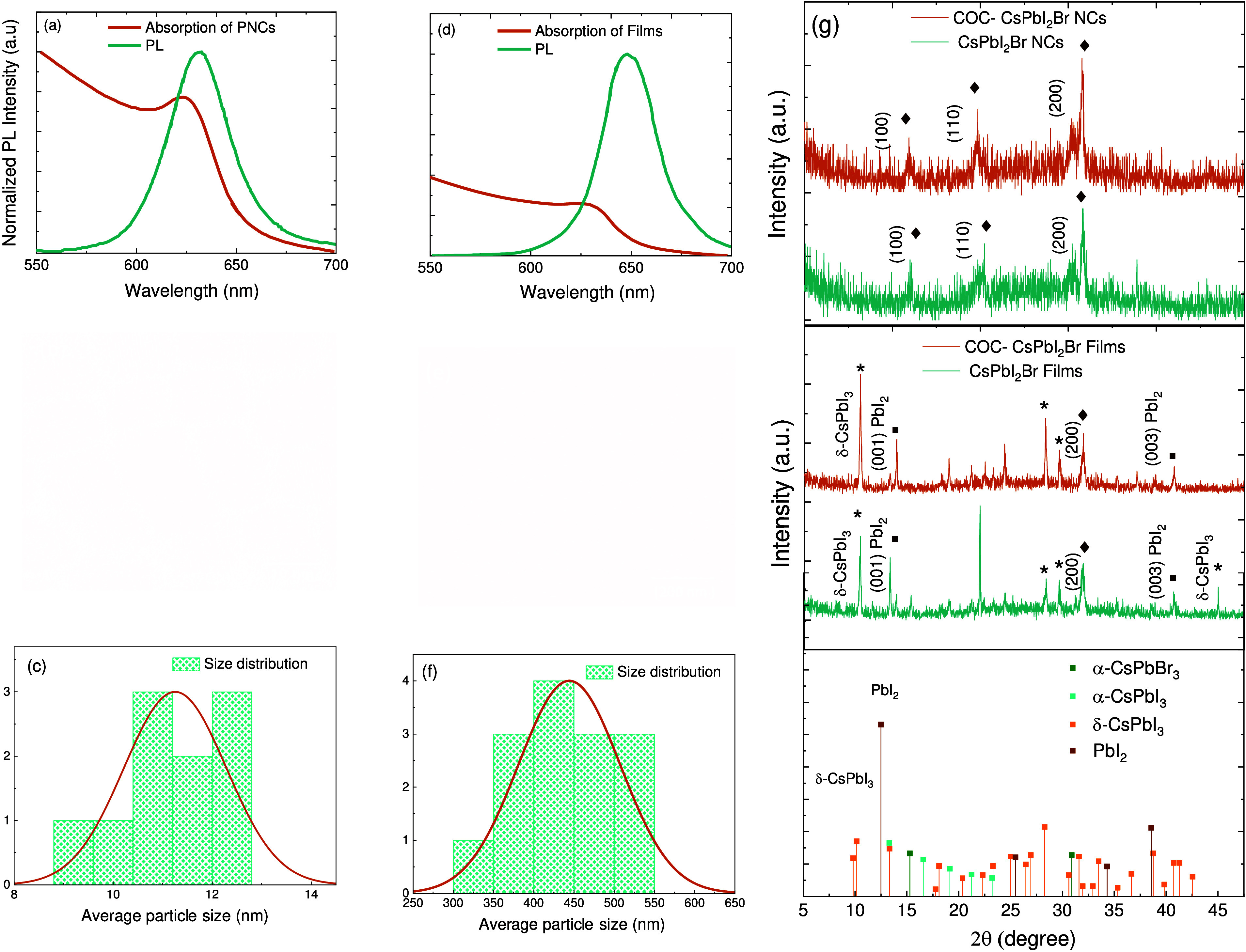
(a) Ultraviolet–visible (UV–vis) absorption and photoluminescence (PL) spectra of CsPbI_2_Br PNCs obtained under air conditions. (b) Transmission electron microscope (TEM) images of CsPbI_2_Br PNCs. (c) Size distribution of CsPbI_2_Br PNCs (11.30 nm in size) from TEM images. (d) UV–vis absorption (PL) spectra of CsPbI_2_Br thin films obtained under air conditions. (e) SEM images of CsPbI_2_Br thin films. (f) Grain size distribution with ∼450 nm CsPbI_2_Br thin films. (g) XRD patterns of the CsPbI_2_Br PNCs and film counterparts before and after encapsulation upon 405 nm illumination with an average power density of 3180 W cm^–2^.

As mentioned in previous reports,^10,37^ iodide ions (I^–^) are photosensitive and can be easily oxidized to form I_2_ in precursor solutions, resulting in nonstoichiometric and iodide vacancy defects (V_I_) in synthesized perovskites. To evaluate the effectiveness of COC in preventing iodide oxidation, we examined the durability of CsPbI_2_Br PNCs and thin films and their encapsulated counterparts. Figure 1g presents the X-ray diffraction (XRD) patterns of CsPbI_2_Br PNCs and CsPbI_2_Br thin films, both before and after encapsulation, under a continuous wave (CW) laser excitation at a wavelength of 405 nm and a power density of 3180 W cm^–2^. Fresh samples of PNCs and COC-encapsulated PNCs showed a cubic structure similar to that of black α-CsPbI_3_ (α-CsPbI_3_ ICSD No. 161481),^11,38−41^ indicated by the peaks at 2θ values of 14.5°, 21.0°, and 30.0° that correspond to the (100), (110), and (200) crystal planes, respectively, with no peaks from PbI_2_ or other impurities, suggesting preserved crystal quality and COC encapsulation on PNC surfaces.^39^ This is derived from the existing data for the CsPbI_2_Br perovskite with a specific crystal structure.^38,42^ In contrast, thin films and COC-encapsulated thin films showed peaks for CsPbBr_3_ cubic and CsPbI_3_ low-crystalline orthorhombic σ-phase at a 2θ of 10.15° and a strong PbI_2_ peak at 2θ values of 12.7° and 38.0°, which correspond to the (001) and (003) crystal planes, respectively,^43,44^ possibly due to iodine and lead ion imbalance.^11^ Although COC-encapsulated thin films had the highest XRD peak intensity and narrowest peak width, PbI_2_ peaks were still observed, indicating that COC does not entirely prevent PbI_2_ byproduct generation. The remnant peaks at 2θ values of 10.15°, 26.5°, and 43.0° correspond to degradation products not included in the perovskite lattice. For instance, we see a prominent PbI_2_ peak at 2θ values of 12.5° and 38.0°, which correspond to the (001) and (003) crystal planes, respectively.

The selective oxidation of halides with lower oxidation potentials (I^–^ < Br^–^ < Cl^–^) is crucial for the movement of halide ions, which ultimately leads to the separation of different phases in perovskite materials^10^ (Figures S3 and S4). Accordingly, time-dependent photoluminescence was implemented to investigate the effect of COC on halide separation and purity of emission color. Panels a and b of Figure 2 show the PL emission during a 50 min continuous illumination on CsPbI_2_Br PNCs and thin films at an excitation wavelength of 405 nm and an average power density of 3180 W cm^–2^ (CW laser); in contrast to those in Figure 1, the sample was never exposed to air and therefore had a shorter emission wavelength. A uniform phase with an initial emission peak at 605 nm of CsPbI_2_Br PNCs is shown in Figure 2a, indicating entropic stabilization of the mixed halide Br/I ions.^45^ Following a 10 min illumination, the green glow at 510 nm and red emission at 606 nm, along with a decreasing PL intensity, emerged due to halide oxidation (e.g., iodine) and ion migration.^10,12^ After 20–30 min, the intensity of red emission decreased and that of green emission increased, with a slight blue shift of 2–3 nm. This indicates the gradual photochemical decomposition of iodine ions and iodine oxidation triggered by the loss of iodine from the lattice, leaving CsI and Pb behind.^46,47^ After 40 min, a distinct green emission with a slight blue shift to 508 nm appeared, while the red emission decreased with a blue shift to 575 nm accompanied by red emission at 685 nm. This trend confirms that CsPbBr_3_ is less susceptible to oxidation than are CsPbI_2_Br and CsPbBI_3_, which is also a sign of iodine sublimation and tribromide phase formation.^12,46^ Accordingly, excess iodine (I^0^) builds up under prolonged illumination, which in turn sublimates as gas from the outermost to the innermost layer nanocrystals, enriching the surface with uncoordinated ions (Pb^2+^, Cs^+^, and I^–^). Under intense light illumination, a surface enriched with uncoordinated ions can continuously decompose as a green glow or be reconstructed as red emission by either photolysis or thermolysis^48^ following the chemical path
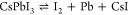
1

**Figure 2:**
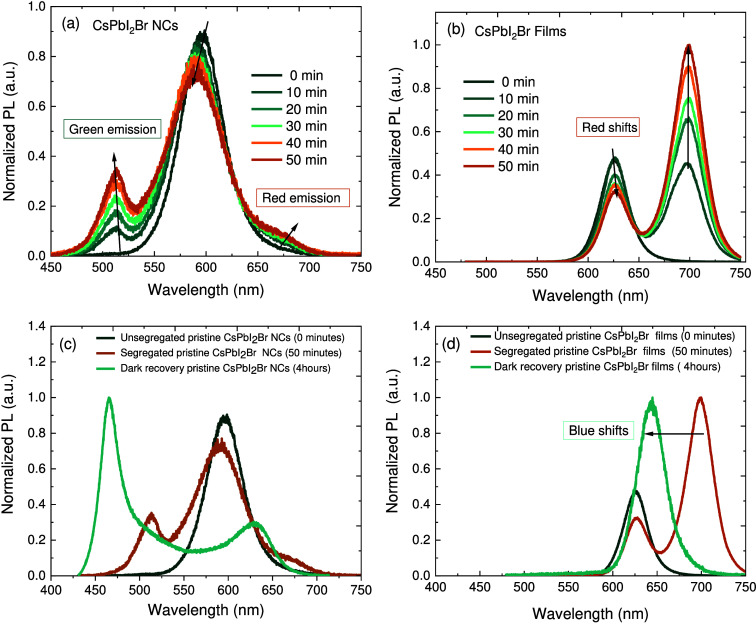
Light-induced phase separation of CsPbI_2_Br PNCs and thin films continuously excited by CW light at 405 nm with a laser power density of 3180 W cm^–2^ during 0, 10, 20, 30, 40, and 50 min illuminations. Evolution of PL for (a) CsPbI_2_Br PNCs and (b) CsPbI_2_Br thin films. (c and d) PL spectra for the initial, light-induced, and dark recovery states of the PNCs and thin films, respectively. The initial spectrum is represented by the dark green line, and the spectrum after illumination for 50 min by the brown line. The turquoise line illustrates the spectrum after illumination for 4 h and the subsequent dark recovery.

In contrast, CsPbI_2_Br thin films in Figure 2b exhibited an initial more red emission peak at 625 nm due to a lower binding energy and weaker quantum confinement with an enlarged crystal size. Following a 10 min illumination, the halide ions begin to migrate, and films were separated into pure red emission at 625 nm and deep red (I-rich) emission at 700 nm, resulting in an inefficient energy transfer process. Unlike that of PNCs, the intensity of pure red emission decreased and that of deep red emission slightly increased, indicating that grain boundaries (GBs) in thin films impair the escape of iodine molecules as triiodide (I_2_ + I^–^ = I_3_^–^) and, thereby, affect the contribution of the hole trap states.^11^ This can also be a sign of the reversible redox (I^–^/I_3_^–^) and (Pb^2+^/Pb^0^) process followed by recombination of photogenerated carriers at low energy to form the CsPbI_3_ phase as represented in the chemical paths from eq 3 to 8. After a 50 min illumination, the red emission intensity (I-rich) peaked and the pure red emission intensity decreased, signifying the gradual depletion of I^–^ and spatial variation in iodide oxidation at grains, leading to the formation of the CsPbI_3_ phase.^11,49−51^ Note that photobrightening of deep red emission can be attributed to stronger photochemical reactions or a photon energy above (a critical wavelength) of ∼520 nm, while photodarkening occurs below this wavelength. Thus, thin films exhibit different halide oxidation processes, with Pb^0^ and I_3_^–^ defects migrating more vertically from the bulk to grain surfaces and grain boundaries than CsPbI_2_Br PNCs (Figures S3 and S4).
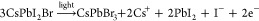
2
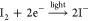
3
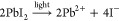
4
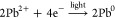
5
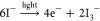
6
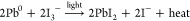
7
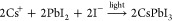
8

To uncover the underlying mechanism leading to photooxidation and the imbalance of Br/I ion migration, we traced the PL emission of PNCs and thin films after restoration for 4 h in the dark. Figure 2c confirms that CsPbI_2_Br PNCs were subjected to an irreversible process of blue-shifted emission and increased green emission with iodine agglomeration, confirming I^–^ photooxidation and sublimation as I_2_. In contrast, the reverse process is shown for CsPbI_2_Br thin films in Figure 2d, resulting in a restored emission spectrum and complete bleaching of the low-energy band at 645 nm due to intermixing Br-rich and I-rich domains driven by entropy stabilization.^11^ This can further prove that the total Gibbs free energy, Δ*G*_total_, in the CsPbI_2_Br thin film is equally attributed to the bulk, Δ*G*_bulk_, and the surface, Δ*G*_surf_. In contrast, surface free energy Δ*G*_surf_ dominates Δ*G*_total_ in the CsPbI_2_Br PNCs due to the higher surface energy.^52,53^
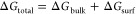
9
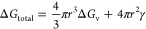
10where *r* represents the particle radius, Δ*G*_v_ is the crystal’s free energy, and γ is the surface tension.

To verify the effectiveness of passivation in inhibiting halide oxidation, we further performed measurements on the same samples after they had been encapsulated in COC. In panels a–c of Figure 3, the PL intensity increased with a slight red shift of 10–12 nm after PNCs were encapsulated. This can be ascribed to a strong coupling involving energy transfer due to the interface effect between PNCs and COC.^54^ It has been suggested that an increased crystal size and a surface effective dielectric constant cause a red shift. The surface ligands in PNCs tend to be eliminated after COC encapsulation, increasing the dielectric constant of the surrounding medium and thus increasing the absorbance and red shift.^55,56^ Previous studies raised the possibility that the electrons localize near the antisite defect site without COC, capturing charge carriers and causing charge recombination. However, the electron distribution tends to be delocalized after the COC coating, implying that localized states are passivated. Thus, COC passivation reduces the interfacial trap state density and strongly binds the COC and PNCs.^56−58^

**Figure 3:**
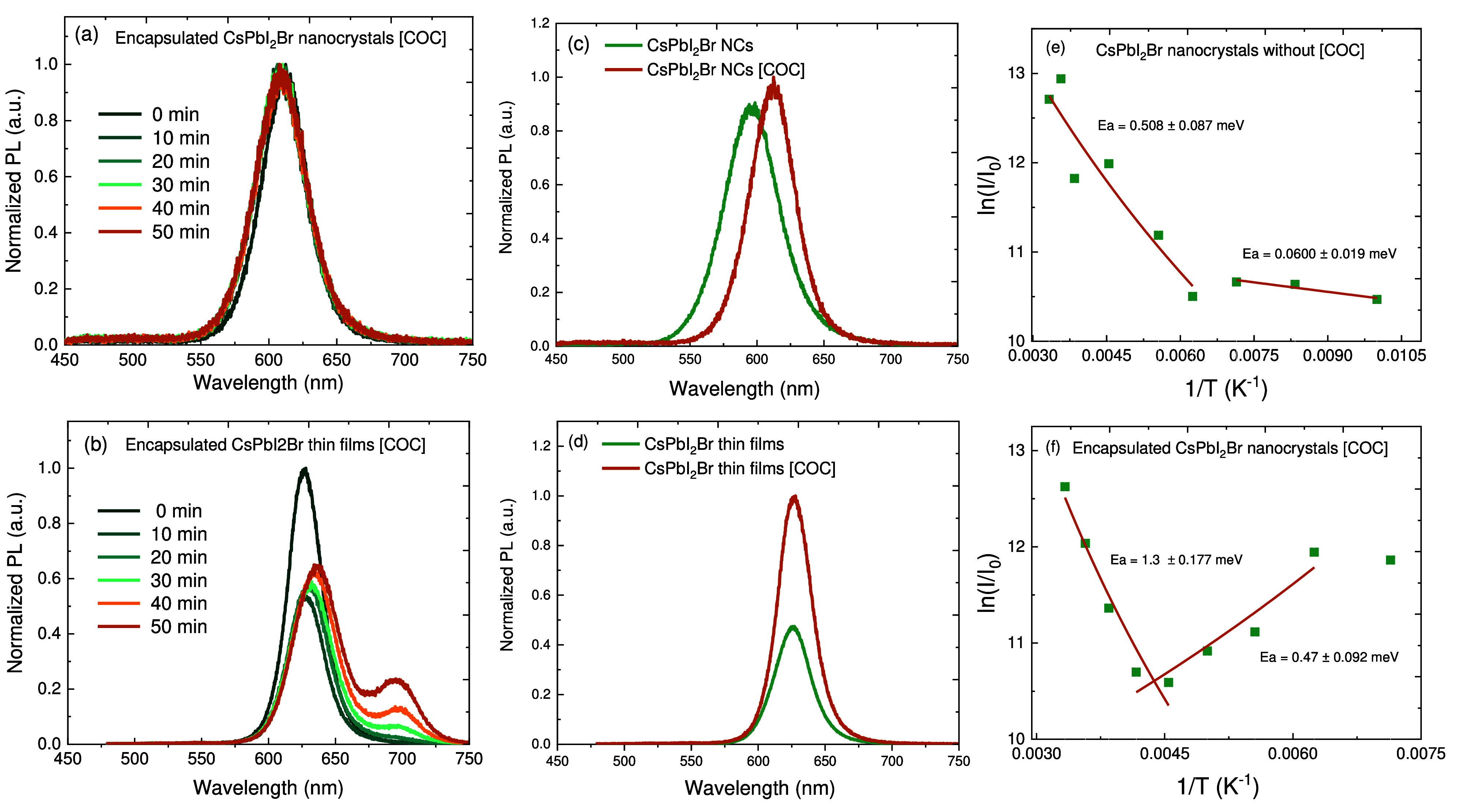
Light-induced phase separation of COC-encapsulated CsPbI_2_Br PNCs and thin films continuously excited by CW light at 405 nm with a laser power intensity of 3180 W cm^–2^ during 0, 10, 20, 30, 40, and 50 min illuminations. (a) Evolution of PL for COC-encapsulated CsPbI_2_Br PNCs. (b) Evolution of PL for COC-encapsulated CsPbI_2_Br thin films. (c) Steady state PL spectra of the PNCs (green line) and the COC-encapsulated CsPbI_2_Br PNCs (orange line). (d) Steady state PL spectra of the thin films (green line) and the COC-encapsulated CsPbI_2_Br thin films (orange line). (e and f) Activation energies of ion migration extracted from Arrhenius plots of the PL of CsPbI_2_Br PNCs and COC-encapsulated CsPbI_2_Br PNCs, respectively. The orange line represents a linear fit to the data (green square).

Unlike the case for PNCs, COC passivation massively inhibited phase separation in the CsPbI_2_Br PNCs and exhibited pure red emission at 615 nm under continuous illumination. The inhibition of halide separation here can be ascribed to the selective reduction of iodine (I^0^) and oxidizes metallic Pb^0^ in a gradual manner.^10,59,60^ On the basis of the XRD data presented in Figure 1g, we suggest that COC inhibits the oxidation of iodide ions of PNCs, thereby decreasing the density of iodine-relevant defects, including iodine vacancies (V_I_) and metallic Pb^0^.^61,62^ Similar to PMMA polymers, COC may passivate these defects, form an equilibrium with surface-bound ions, and maintain charge neutrality instead of oxidation. It may also suppress the nearest neighbor hopping mechanism in mixed halide (Br/I) ions,^63−66^ which more likely occurs between adjacent lattice sites and halide vacancies (V_X_) that serve as the pathway for halide migration.^64,66^

Previous density functional theory calculations^57,64,67^ raise the possibility that electrons are transferred from PNCs to COC, reducing the level of self-doping and transforming PNC conductivity from n-type to balanced charge carriers. Specifically, Pb atoms acquire an overall positive charge as uncoordinated Pb^2+^ ions when vacancies are produced. The electrostatic attraction of photoexcited electrons into Coulomb trap sites V_X_ also facilitates a nonradiative pathway and phase separation. These vacancies can also induce n-type behavior in PNCs, limiting the performance of optoelectronic devices. Therefore, the COC might fill these vacancies through a strong interaction with Pb^2+^ ions, eliminating electron traps and improving the stability.

In contrast, Figure 3b shows that CsPbI_2_Br thin films still exhibited phase separation after encapsulation, where an initial emission peak at 625 nm shifted to the red with PL intensity reduction within 10–30 min. This can be attributed to an entropic destabilization in which the lattice strain energy in thin films is still higher due to the presence of multisurface defects compared to that of PNCs;^11^ therefore, the Gibbs free energies (Δ*G*) for the half-reactions I^0^ → I^–^ and Pb^0^ → Pb^2+^ are not sufficiently reduced.^11,59,60^ Although all perovskites have similar defects, reducing the grain size of perovskites to a few nanometers can limit the degree of excited charge carrier spatial freedom, inhibiting the diffusion of halide ions into halide vacancies V_X_ in mixed halide perovskites.^64,67^ Moreover, after illumination for >50 min, halide ion migration accelerated phase separation into the Br-rich domain and I-rich domain with red shifts from 625 to 650 nm and 700 to 705 nm, respectively. Unlike pristine thin films (Figure 3d), COC enhanced the brightness in both Br-rich and I-rich domains due to the reduced charge carriers being funneled from mixed to separated phases^54^ (Figures S3 and S4).

The instability of mixed halide perovskites has been attributed to the low activation energy of halide ion migration, which facilitates more halide vacancy defects. To better understand this, temperature-dependent PL emission was implemented to determine the ion migration activation energy (*E*_a_) (Figure S5). In panels e and f of Figure 3, the Arrhenius curves of the PNCs and encapsulated PNCs consist of two linear regions. The PNCs showed a lower *E*_a_ of 0.50 meV above 160 K and an *E*_a_ of 0.060 meV below 160 K. These values are close to those from the measurements provided in Table 1 of the Supporting Information. In the case of encapsulated PNCs, the low-temperature linear region below 200 K showed an *E*_a_ of 0.47 meV due to electron/hole transport from photoconductivity under illumination. However, the high-temperature linear region above 200 K exhibited a higher *E*_a_ of 1.3 meV, corresponding to active ion migration, which helped to increase conductivity exponentially.^68^ A similar trend was also found in thin films in which *E*_a_ increased from 20 to 29 meV after being encapsulated with COC.^54^ On the basis of the results presented above, we confirm that encapsulation can effectively increase the *E*_a_ of halide ions, which is linked to inhibiting or slowing its migration.

Time-resolved PL (TRPL) measurements were employed to uncover the charge transport in these materials, as shown in Figure 4a–d. TRPL spectra of all samples showed a biexponential decay (Figures S6 and S7). The short-lived components of the PL decay curves arise from surface recombination in films^69,70^ and to excitons in nanocrystals,^71^ while the longer-lived components are usually ascribed to emission from bulk recombination in the case of films^69,70^ and shallow surface traps in the case of nanocrystals.^71,72^ The fast decay lifetime (τ_1_) and slow decay lifetime (τ_2_) were 1.07 and 5.48 ns, respectively, for CsPbI_2_Br PNCs and 1.29 and 8.70 ns, respectively, for CsPbI_2_Br thin films. The fast decay lifetime (τ_1_) and slow decay lifetime (τ_2_) were 1.30 and 13.88 ns, respectively, for the encapsulated CsPbI_2_Br PNCs and 2.93 and 18.64 ns, respectively, for the encapsulated CsPbI_2_Br thin films. The prolonged lifetimes of the encapsulated PNCs and thin films are associated with a decrease in the electronic trap density and increased radiative recombination rates.^59,73^ Notably, the average carrier lifetime (τ_avg_ at 600 and 612 nm) was increased from 2.19 to 3.93 ns before light soaking and from 1.32 to 10.51 ns after light soaking when PNCs were being encapsulated (Figure S6). Similarly, the average carrier lifetime (τ_avg_ at 645 nm) was increased from 3.68 to 16.50 ns before light soaking and from 2.73 to 23 ns after light soaking after the thin film was encapsulated (Figure S7). The increase can be attributed to the inhibition of highly mobile ions (I^–^) resulting from internal iodine migration after hole-induced iodine oxidation.^62,74−77^

**Figure 4:**
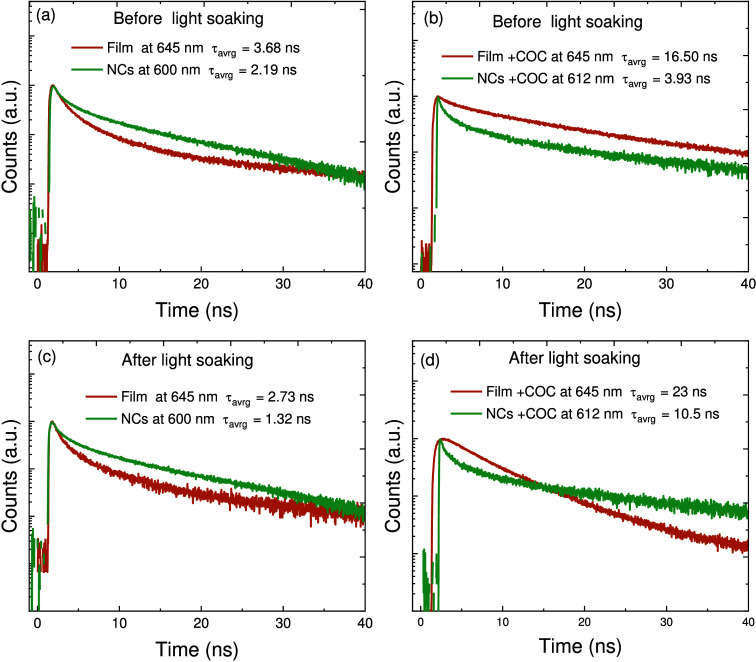
Time-resolved photoluminescence (TRPL). (a) Pristine CsPbI_2_Br PNCs and CsPbI_2_Br thin films before a 50 min illumination. (b) COC-encapsulated CsPbI_2_Br PNCs and CsPbI_2_Br thin films before a 50 min illumination. (c) CsPbI_2_Br PNCs and CsPbI_2_Br thin films after a 50 min illumination. (d) COC-encapsulated CsPbI_2_Br PNCs and CsPbI_2_Br thin films after a 50 min illumination.

In summary, we have monitored halide ion mobility through photoinduced halide separation and dark recovery after reducing the dimensionality of mixed halide perovskites from films to nanocrystals. Our results demonstrate a strategy for achieving the long-term stability of the pure red emission using mixed halide CsPbI_2_Br PNCs encapsulated in polymer COC. Experimental observations showed that COC not only passivates uncoordinated surface (Pb and I/Br) defects and reduces I^–^ ion species but also increases the energy of formation of halide vacancies. Moreover, the encapsulation of these materials can drive electron transfer from the CsPbI_2_Br PNCs to the COC, which extends the lifetime of charge carriers and reduces nonradiative recombination rates. These results help us to understand the causes of photodecomposition and highlight the function of halide vacancy passivation in increasing the stability of mixed halide CsPbI_2_Br PNCs compared to that of their film counterparts, thus providing a better approach for fabricating highly efficient pure red LEDs using NCs and/or polymer matrices.

## Supporting Information Available

Sample synthesis and optical characterization, including TEM images of nanocrystals, and outlines of the photooxidation mechanism of CsPbI_2_Br, supported by spectroscopic data, including temperature-dependent photoluminescence, time-resolved decay curves, and structural data (PDF)
