# Supplementary material for: Inhibiting the Appearance of Green Emission in Mixed Lead Halide Perovskite Nanocrystals for Pure Red Emission
Source: Nano Lett. 2024 Sep 23;24(39):12045–53. doi: 10.1021/acs.nanolett.4c01565 (PMC11450971; doi:10.1021/acs.nanolett.4c01565)
Supplement: Supplementary file 1 — nl4c01565_si_001.pdf [file nl4c01565_si_001.pdf]

Inhibiting the Appearance of Green emission in Mixed Lead halide Perovskite Nanocrystals for Pure Red Emission

Mutibah Alanazi[1], Ashley R. Marshall[1,2], Yincheng Liu[1,3], Jinwoo Kim[1], Shaoni Kar[1,2], Henry J. Snaith[1], Robert A. Taylor[1], and Tristan Farrow*[4,1]

[1]Clarendon Laboratory, Department of Physics, University of Oxford, Parks Road, Oxford, OX1 3PU, U.K.
[2]Helio Display Materials Ltd., Wood Centre for Innovation, Oxford, OX3 8SB
[3]Institute of Materials Research and Engineering, Agency for Science, Technology and Research (A*STAR), 2 Fusionopolis Way, Singapore 138634, Singapore
[4]NEOM Education, Research and Innovation Foundation, and NEOM University, Tabuk 49643-9136, Saudi Arabia
*Corresponding authors: tristan.farrow@cantab.net

Experimental Methods

Synthesis

Nanocrystal Synthesis: PbX_2 powder (0.2 mmol), CsX powder (0.2 mmol), OA (0.5 mL), OAm (0.25 mL) and DMAI (for the A-site modified NCs) were added to 5 mL of dimethylacetamide (DMAc) and stirred to dissolve for 60 mins at room temperature. A portion (3 mL) of the obtained mixture was swiftly injected into 30 mL chlorobenzene or chloroform under vigorous stirring. After stirring, the resulting solution was mixed with methyl acetate in a 1:2 ratio (v/v) and centrifuged for 5 min at 7500 revolutions per minute (rpm). After centrifugation, the supernatant was discarded, and the precipitate was dispersed in hexane. Stable CsPbI2Br NC ink ($\sim$10 mg mL^{-1}) was obtained by removing larger particles after centrifuging the crude dispersion for 5 min at 3000 rpm. Further washing (repetition of fast and slow centrifugation steps) can be done for device application.

Film Fabrication (solution processing):The ITO substrates underwent a cleaning process involving sequential sonication in DECON-90, DI water, acetone, and IPA for a duration of 15 minutes each. Following that, the substrates were subjected to UV-ozone treatment for a further 15 minutes. The films utilized for fundamental characterisation and devices were fabricated using the spin-coating technique on ITO/PEDOT:PSS substrates.The PEDOT:PSS solution was subjected to a spin-coating process in ambient conditions, with a rotation speed of 5000 rpm for a duration of 40 seconds. Subsequently, the coated film was subjected to annealing at a temperature of 150 °C for a period of 12 minutes. Afterwards, the specimen was transferred to the glovebox antechamber and left to undergo a drying process for a duration of 20 minutes. Then, NC ink was spin-coated for 40 seconds at 1500 rpm in the glovebox. Any residual solvent was evaporated at 50 °C for a duration of 10 minutes.

Optical Characterization

Photoluminescence spectroscopy: The substrate was mounted in a Janis continuous-flow helium cryostat and the temperature was varied between 4.2 K and room temperature by a Lakeshore 331 temperature controller. Optical characterization was conducted using a confocal micro-photoluminescence set-up. The pump was a continuous wave wavelength laser source (405 nm) and a pulsed variable-wavelength laser source (NTK SuperK Extreme) operating at a maximum frequency of 78 MHz. A 100× magnification apochromatic objective was used in the confocal set-up to focus the pump beam to a spot size of $\sim 1\,\mu m^2$ and to collect the luminescence. The spectra data of PL spectroscopy was collected using a SpectraPro HRS spectrometer.

Time Resolved Photoluminescence (TRPL) spectroscopy: TRPL measurements were carried out with a time-correlated single photon counting system (PicoHarp 300) coupled with 2 single photon avalanche diodes (MPD PDM) and the time constants were determined using EasyTau.

UV-Vis absorption : Separately,absorbance spectra were measured with a Varian Cary 300 Bio UV-Visible spectrophotometer with a 50×50 mm reflective neutral density filters with an optical density of 3.0 (made out of UV fused silica).

4.2.4 X-ray diffraction spectroscopy: XRD diffractograms were obtained using a Panalytical X'Pert Pro X-ray diffractometer with a Cu-alpha source ($\lambda = 1.54$ Å).

Transmission Electron Microscope (TEM): PNC solutions ($\sim$50 mg/mL) were diluted to about 10 mg/mL (as indicated by their optical densities measured on a UV-visible spectrometer) in 9:1 volume ratio of toluene: hexane solvent. This was then drop cast onto a carbon-coated copper TEM mesh-grid and the samples left to dry in the glovebox antechamber for about an hour. The TEM imaging was then done on a JEOL JEM-2100 microscope operating an LaB6 source and equipped with

an Oxford instrument 80 mm thin window EDS detector and a Gatan Orius CCD camera. The ImageJ software was using to analysis the crystal Size.

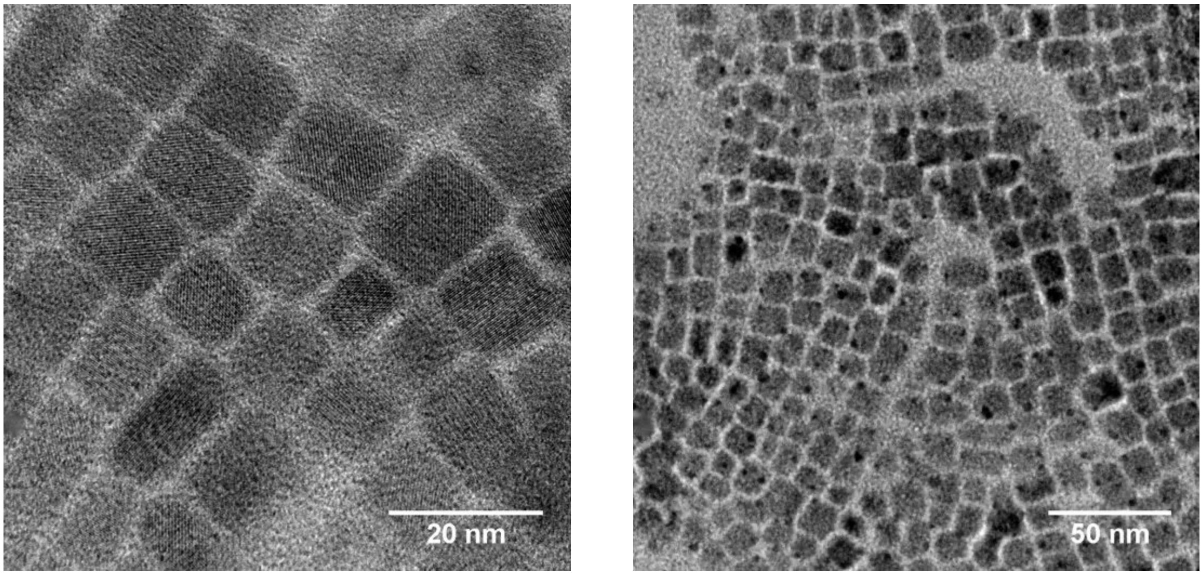

Figure S1. Transmission electron microscopy (TEM) images depicting nanocrystals composed of CsPbI2Br.

The photo-oxidation mechanism of $CsPbI_2Br$ nanocrystals and thin films.

The absence of a green glow in pristine thin films is due to the accumulation of Pb^0 and I_3^- defects, which increase the lattice energy of pristine thin films, and then diffuse to grain boundaries, forming an I-rich phase due to the presence of multiple surface defects in the thin film compared to nanocrystals (e.g. dangling bonds and uncoordinated (Pb^{2+} ions). This can further refer to the fact that the diffusion of both Pb^0 and I_3^- defects in thin films has a higher propensity to migrate from the bulk to the surfaces of grains and grain boundaries along a vertical pathway than pristine nanocrystals. The inter-diffusion rates for this migration range approximately between 10^{19} to 10^{20} m^2s^{-1}[1]. In specific, upon prolonged illumination, the thin films decompose into CsI,PbI_2, and $PbBr_2$ which can passivate defects and grain boundaries to suppress the non-radiative recombination[2]. Mobile interstitial iodine (I^0) is attracted by charge carriers accumulated at low energy regions (I-rich domains), whereas the (Br_x^-) and (I^0/ I^-) interdiffuse to reduce lattice strain. In this regard, we deduce that the band gap difference between I-rich and Br-rich areas determines the threshold size or the intensity that causes phase segregation. In addition, the lattice strain determines the preferential direction for inter-diffusion of both charge carriers and the iodine mobility, which are responsible for final halide distribution.

The activation energy (Ea) measured in this work		
	$CsPbBrI_2$ nanocrystals	$CsPbBrI_2$ thin films
Pristine Without COC	20.6 meV	20.8 meV[4]
Encapsulated With COC	39.3 meV	29 meV[4]
The activation energy (Ea) of various lead Halide Material		
	Process	The activation energy(Ea)
$CsPb(I_{0.5}Br_{0.5})_3$ Colloidal NCs	Photo-decomposition	62 kJ/mol[5]
$CsPb(Cl_{(1-x)}Br_x)_3$ Colloidal NCs	Halide Exchange	63 kJ/mol[6]
PbI_2 thin film	Photo-decomposition	16 kJ/mol[7]

Table 1. The activation energy of pristine $CsPbI_2Br$ and encapsulated $CsPbI_2Br$ nanocrystals and films compared to the previous activation energy for various mixed halide compositions.

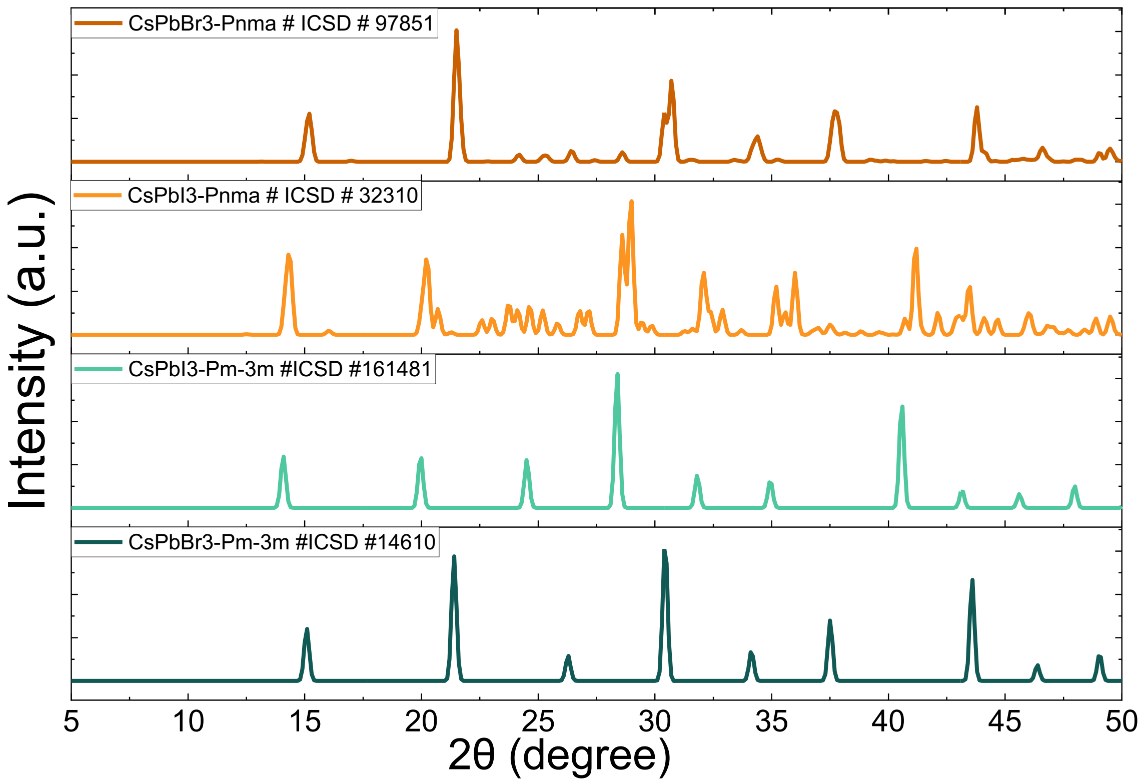

Figure S2. The reference patterns of the orthorhombic, cubic, and tetragonal phases of $CsPbBr_3$ and $CsPbI_3$ obtained in the Inorganic Crystal Structure Database (ICSD).

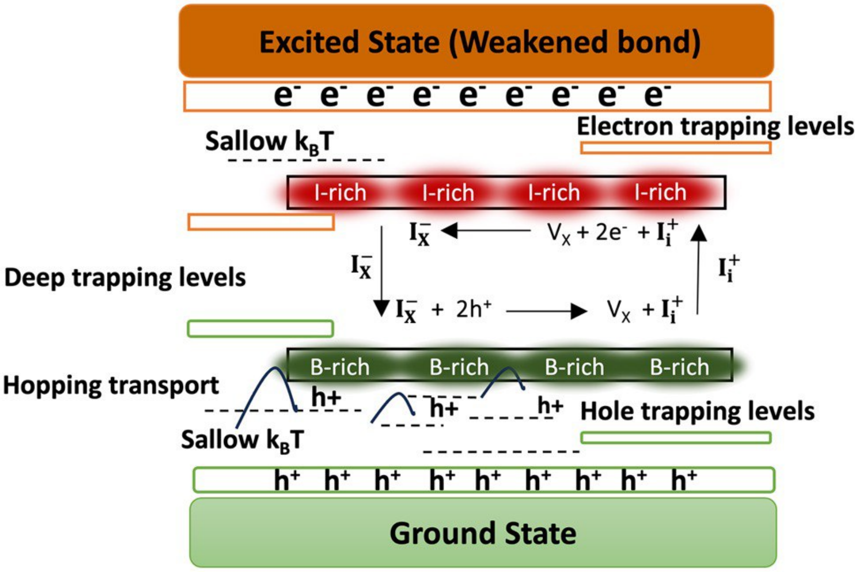

Figure S3. Schematic illustrating the photoinduced halide oxidation mechanism for mixed halide perovskites in the form of $CsPbI_2Br$ nanocrystals and thin films. The photogenerated holes (2 h^+) oxidize the lattice iodide ions (I_x^-), leading to the formation of iodine (I_2) and / or triiodide (I_3^-), either in the bulk (I^*) or at surfaces (I^0)[3]. If it forms in bulk, the (I_2) migrate faster and are then expelled from the lattice, where uncoordinated Pb^{2+} and Cs^+ ions accumulate at the nanocrystal surface. Meanwhile, the lattice bromine ions (Br_x^-) drift or diffuse into the Br-rich region and reconstruct the $CsPbBr_3$ instead of photo-decomposition due to a higher oxidation potential than for the lattice iodide (I_x^-).

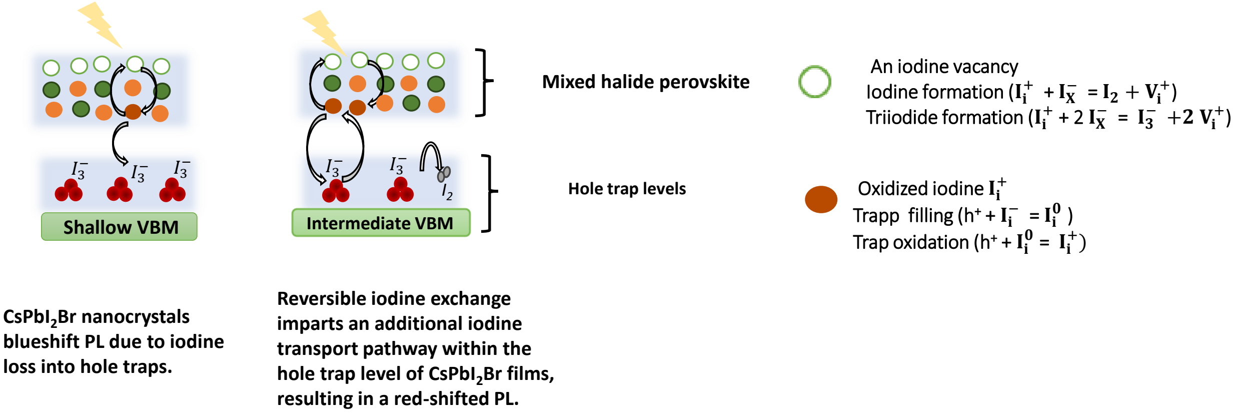

Figure S4. Schematic illustrating the photoinduced halide oxidation mechanism of mixed halide perovskites in the form of $CsPbI_2Br$ nanocrystals and $CsPbI_2Br$ thin films.

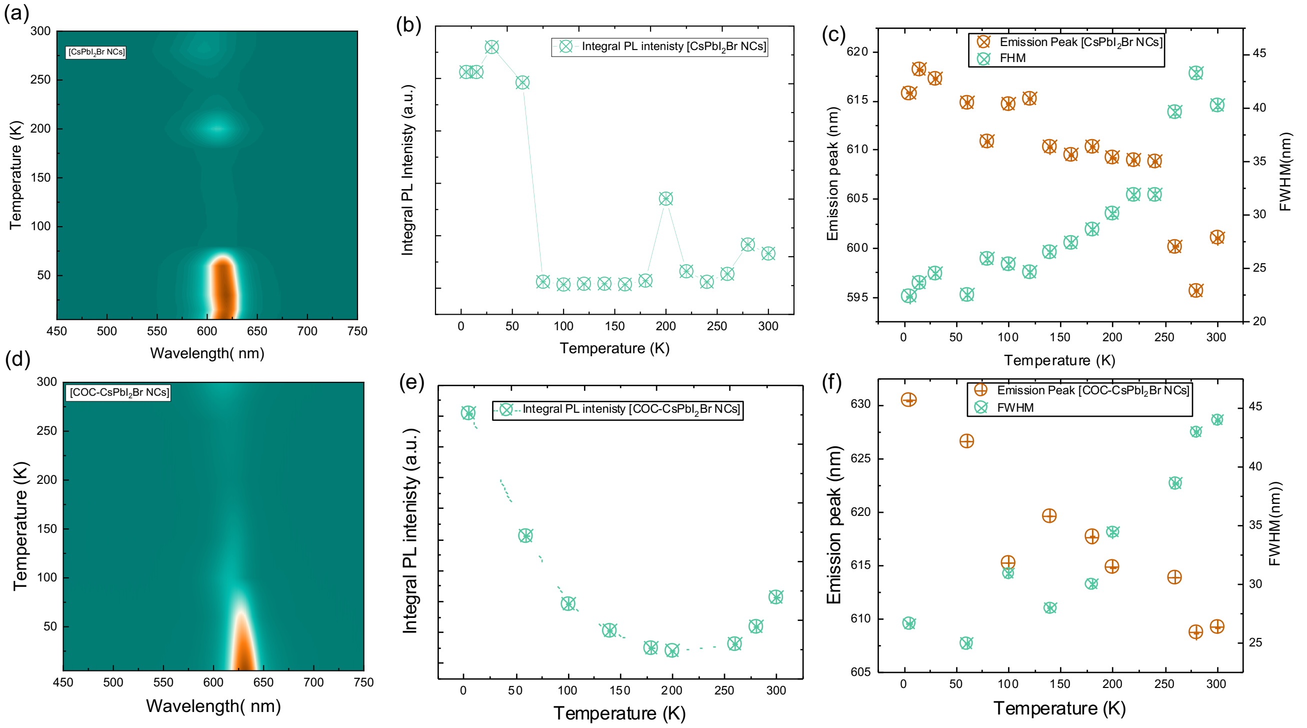

Figure S5. Temperature dependent photoluminescence of $CsPbI_2Br$ nanocrystals and their COC-encapsulated counterparts (a-d) The PL spectra of a 2D -colour plot of the $CsPbI_2Br$ NCs and encapsulated counterparts at temperatures ranging from 5 to 297 K. (b-e) The integrated PL intensity as a function of temperature for $CsPbI_2Br$ nanocrystals and COC-encapsulated $CsPbI_2Br$ (d-e)The emission peak and FWHM as a function of temperature for nanocrystals and COC-encapsulated $CsPbI_2Br$,respectively.

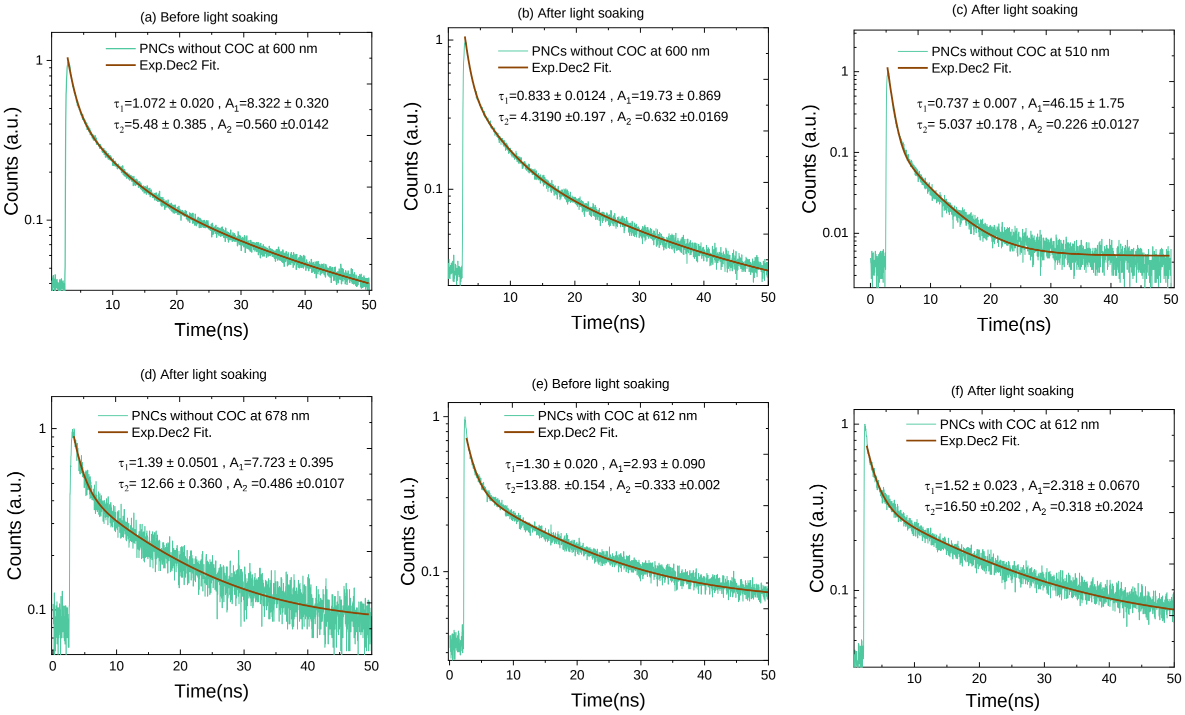

Figure S6. Time decay parameters of $CsPbI_2Br$ nanocrystals and their COC-encapsulated counterparts before and after 50 minutes of a blue light illumination at a wavelength of 450 nm at a laser repetition rate of 7.8 MHz for PNC with COC and 16 MHz for PNCs without COC, respectively. The fitted TRPL decay parameters were extracted using the equation:

$$f(t) = A_1 \exp(-t/\tau_1) + A_2 \exp(-t/\tau_2) + y_0,$$

Where τ_1 refers to short-lived PL emission, and τ_2 represent the long-lived lifetime

References

1. Akriti *et al.* Layer-by-layer anionic diffusion in two-dimensional halide perovskite vertical heterostructures. *Nat. Nanotechnol.* **16**, 584–591 (2021).
2. Chen, Q. *et al.* Controllable self-induced passivation of hybrid lead iodide perovskites toward high performance solar cells. *Nano letters* **14**, 4158–4163 (2014).
3. Kerner, R. A., Xu, Z., Larson, B. W. & Rand, B. P. The role of halide oxidation in perovskite halide phase separation. *Joule* **5**, 2273–2295 (2021).
4. Alanazi, M. *et al.* Stability of mixed lead halide perovskite films encapsulated in cyclic olefin copolymer at room and cryogenic temperatures. *The J. Phys. Chem. Lett.* **14**, 11333–11341 (2023).
5. Brennan, M. C. *et al.* Photolysis of mixed halide perovskite nanocrystals. *ACS Energy Lett.* **8**, 2150–2158 (2023).
6. Scharf, E. *et al.* Ligands mediate anion exchange between colloidal lead-halide perovskite nanocrystals. *Nano Lett.* **22**, 4340–4346 (2022).
7. Dawood, R., Forty, A. & Tubbs, M. The photodecomposition of lead iodide. *Proc. Royal Soc. London. Ser. A. Math. Phys. Sci.* **284**, 272–288 (1965).

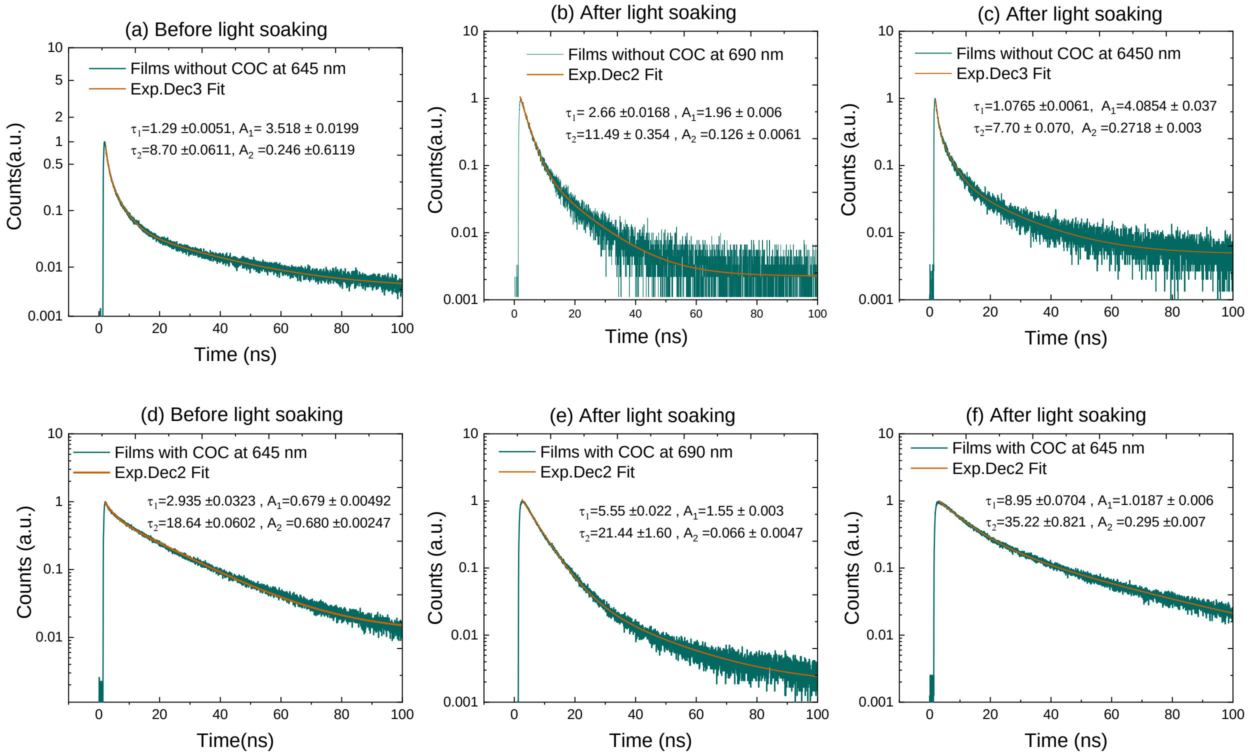

Figure S7. Time decay parameters of $CsPbI_2Br$ thin films and their COC-encapsulated counterparts before and after 50 minutes of a blue light of illumination at a wavelength of 450 nm and a 8.3 MHz laser repetition rate. The fitted TRPL decay parameters were extracted using the equation:

$$f(t) = A_1 \exp(-t/\tau_1) + A_2 \exp(-t/\tau_2) + y_0,$$

Where τ_1 refers to short-lived PL emission , and τ_2 represent the long-lived lifetime
